# Longitudinal investigation of the swine gut microbiome from birth to market reveals stage and growth performance associated bacteria

**DOI:** 10.1186/s40168-019-0721-7

**Published:** 2019-07-30

**Authors:** Xiaofan Wang, Tsungcheng Tsai, Feilong Deng, Xiaoyuan Wei, Jianmin Chai, Joshua Knapp, Jason Apple, Charles V. Maxwell, Jung Ae Lee, Ying Li, Jiangchao Zhao

**Affiliations:** 1 Department of Animal Science, University of Arkansas, Fayetteville, AR USA; 2 Farm Animal Genetic Resources Exploration and Innovation Key Laboratory of Sichuan Province, Sichuan Agricultural University, Chengdu, Sichuan China; 3 Agricultural Statistics Laboratory, University of Arkansas, Fayetteville, AR USA

**Keywords:** Swine gut microbiome, Dynamics, FMT, Growth stage and performance

## Abstract

**Background:**

Despite recent advances in the understanding of the swine gut microbiome at different growth stages, a comprehensive longitudinal study of the lifetime (birth to market) dynamics of the swine gut microbiome is lacking.

**Results:**

To fill in this gap of knowledge, we repeatedly collected a total of 273 rectal swabs from 18 pigs during lactation (day (d) 0, 11, 20), nursery (d 27, 33, 41, 50, 61), growing (d 76, 90, 104, 116), and finishing (d 130, 146, 159, 174) stages. DNA was extracted and subjected to sequencing with an Illumina Miseq sequencer targeting the V4 region of the 16S rRNA gene. Sequences were analyzed with the Deblur algorithm in the QIIME2 package. A total of 19 phyla were detected in the lifetime pig gut microbiome with *Firmicutes* and *Bacteroidetes* being the most abundant. Alpha diversity including community richness (e.g., number of observed features) and diversity (e.g., Shannon index) showed an overall increasing trend. Distinct shifts in microbiome structure along different growth stages were observed. LEfSe analysis revealed 91 bacterial features that are stage-specific. To validate these discoveries, we performed fecal microbiota transplantation (FMT) by inoculating weanling pigs with mature fecal microbiota from a growing stage pig. Similar stage-specific patterns in microbiome diversity and structures were also observed in both the FMT pigs and their littermates. Although FMT remarkably increased growth performance, it did not change the overall swine gut microbiome. Only a few taxa including those associated with *Streptococcus* and *Clostridiaceae* were enriched in the FMT pigs. These data, together with several other lines of evidence, indicate potential roles these taxa play in promoting animal growth performance. Diet, especially crude fiber from corn, was a major factor shaping the swine gut microbiome. The priority effect, i.e., the order and timing of species arrival, was more evident in the solid feed stages.

**Conclusions:**

The distinct stage-associated swine gut microbiome may be determined by the differences in diet and/or gut physiology at different growth stages. Our study provides insight into mechanisms governing gut microbiome succession and also underscores the importance of optimizing stage-specific probiotics aimed at improving animal health and production.

**Electronic supplementary material:**

The online version of this article (10.1186/s40168-019-0721-7) contains supplementary material, which is available to authorized users.

## Background

The advent of next-generation sequencing has dramatically expanded our understanding of the roles that gut microbiome plays in human health and diseases. Given the fact that pigs serve as an important protein source as well as a biomedical model for diseases in humans, the swine gut microbiome has drawn increasing attention. The correlation between swine gut microbiome and animal health and production during critical growth stages has been characterized in several studies [1–6]. High morbidity (e.g., diarrhea) and mortality rate during weaning, attributed to various stresses, reduced gut barrier function, and increased pathogen infection, have been associated with an imbalanced gut microbiome (dysbiosis) which lead to remarkable losses in the swine industry. Modulation of the swine gut microbiome via probiotics and/or prebiotics to maintain a healthy microbiome has been a promising means of preventing pathogens and promoting beneficial bacteria abundances [7]. Particularly, bacterial taxa such as *Christensenellaceae*, *Oscillibacter*, *Defluviitaleaceae incertae sedis*, *Cellulosilyticum*, and *Corynebacterium* have been positively related to feed efficiency [8], which is critical for the swine industry. Recent studies have also filled some knowledge gaps of the swine gut microbiome, with respect to the biogeography of the gastrointestinal tract [5], adiposity [3], digestibility [4], and growth performance [6].

In addition, some larger scale studies have investigated the swine gut microbiome in greater depth. Xiao and colleagues [9] sequenced the fecal metagenomes of 287 pigs from France, Denmark, and China and identified 7.7 million non-redundant genes representing 719 metagenomic species. Interestingly, 96% of the functional pathways found in the human gene catalogue are present in the swine gut microbiome gene catalogue, confirming the importance of pigs as human biomedical models [9]. Lu et al. [10] analyzed the swine gut microbiomes at weaning, week 15, and off-test in over 1000 pigs. They identified two enterotypes at each time point and found that the ones at the two later time points were associated with back fat thickness [10]. In another study, De Rodas and colleagues [11] characterized longitudinal changes of the swine gut microbiome along different anatomical sites over seven time points. They found that the introduction of solid feed between days 21 and 33 had greater overall impact on bacterial community structure than age, solid feed type, and environment did [11].

Although these studies have remarkably expanded our understanding of the swine gut microbiome, they were either cross-sectional or sporadic with large sampling intervals [12, 13]. Many key ecological questions still remain unanswered. For example, how does the swine gut microbiome change from birth to market across all the different growth stages? What are the key drivers shaping the swine gut microbiome during these stages? Which gut microbiota members are residents of the swine GI tract that persist in the gut across age and which ones are passengers that only appear for a short period of time? How do these members correlate with animal health and growth performance? To answer these questions, a comprehensive, longitudinal study of the swine gut microbiome spanning every growth stage from birth to market is imperative.

In this study, we addressed several of these important questions by characterizing the longitudinal changes in the swine gut microbiome from farrow to market covering the lactation, nursery, growing, and finishing stages. We observed significant changes in the swine gut microbiome along these different stages and identified stage- and growth-associated bacterial taxa. To validate our discoveries, we inoculated weaning pigs with mature gut microbiota from a growing stage pig. Similar patterns of changes in the swine gut microbiome were observed in the control group, i.e., pigs without fecal microbiota transplantation (FMT). Although FMT significantly increased the growth performance of the pigs, it did not significantly change the overall gut microbiome structure of the recipients immediately after inoculation, confirming the stage-specificity of the swine gut microbiome likely attributable to gut physiology and diet.

## Methods

### Study design and animals

#### Animal trial 1 (test trial)

Pigs were managed according to the Institutional Animal Care and Use Committee (IACUC) approved protocol #19017. Rectal swabs were collected from 18 pigs (PIC29*380) born from 3 sows (second parity) from the University of Arkansas-Division of Agriculture Swine Research Unit. Among these pigs, 17 were followed throughout all the different growth stages. The piglets were sow fed during lactation till weaning at day (d) 20, when they were transferred to an offsite nursery facility. Piglets were stratified by sow with two littermates housed in a pen. On d 61, pigs were moved to a growing and finishing facility together with their penmates. All pigs were fed with a seven-feeding-phase regime including three nursery phases (NP1: d 20–33; NP2: d 33–50; NP3: d 50–61), two growing phases (GP1: d 61–90; GP2: d 90–116), and two finishing phases (FP1: d 116–146; FP2: d 146–174). All diets were antibiotic-free, and dietary nutrients met or exceeded the NRC (2012) recommendation. Individual pig body weight (BW) and rectal swabs were collected on d 0, 11, and 20 during lactation, at the end of each phase during nursery period, in the middle and the end of each phase during growing/finishing period (Additional file 1: Table S1).

#### Animal trial 2 (validation trial)

To validate the discoveries from trial 1, a total of 24 weaned pigs (PIC29*380) were selected from the University of Arkansas-Division of Agriculture Swine Research Unit (IACUC protocol #19024). Pigs were blocked by sow and assigned to one of four pens in an onsite nursery facility (6 pigs per pen). On weaning day (d 21 of age), half of the pigs (*n* = 12) were treated with fecal microbiota transplantation (FMT). For the FMT, freshly defecated feces from a mature healthy pig from growing stage phase 2 were collected from the anus after rectal massage and were then transferred into a sterile Whirl-Pak® filter bag with a pore size of 0.33 mm (Nasco Fort Atkinson, WI) filled with 20% glycerol in PBS. Bacterial cells were detached from fecal matter after mixing at high speed for 2 min using a Stomacher™400 (Seward Ltd, West Sussex, UK). Filtrates were then transferred into 50-ml conical tubes and stored at − 80 °C freezer. Each pig was gavaged with 3 ml filtrates for two consecutive days (d 21 and d 22). Pigs were fed a total of 8 feeding phase regimes in this trial: three nursery phases (NP1: d 21–29; NP2: d 29–42; NP3: d 42–61), two growing phases (GP1: d 61–84; GP2: d 84–99), and three finishing phases (FP1: 99–138; FP2: d 138–159; FP3: 159–183). All diets were antibiotic-free and met or exceeded NRC (2012) recommendation on nutrient requirement for each stage of pigs. Individual pig BW and rectal swab were collected at weaning and at the end of each phase.

### Sample collection, DNA extraction, and sequencing

A total of 273 rectal swabs (Puritan®Opti-Swab® Liquid Amies Collection & Transport System; Puritan LLC, Guilford, ME) were collected from 17 pigs repeatedly during lactation (d 0, 11, 20), nursery (d 27, 33, 41, 50, 61), growing (d 76, 90, 104, 116), and finishing (d 130, 146, 159, 174) stages in animal trial 1, with two more samples collected from the 18th pig that died during lactation stage. In trial 2, a total of 246 rectal swabs were collected from 24 pigs repeatedly at the end of lactation (d 21), nursery (d 22, 23, 29, 42, and 61), growing (d 84 and 99), and finishing (d 138, 159, and 183) periods (Additional file 1: Table S1) to validate the findings from trial 1. These swabs were stored at − 80 °C until DNA extraction was performed.

A total of 200 μL fecal swab solution was used for DNA extraction with PowerLyzer PowerSoil DNA Isolation Kit (Qiagen, Hilden, Germany) according to the manufacturer’s protocol. Extracted DNA was quantified using NanoDrop (Thermo Fisher Scientific, Wilmington, DE, USA) and diluted to 10 ng/μL with DNase- and RNase-free water. Libraries were constructed according to published protocol [14]. Briefly, the V4 region of the bacterial 16S rRNA gene was amplified using universal primers (F: 5′-GTGCCAGCMGCCGCGGTAA-3′ and R: 5′-GGACTACHVGGGTWTCTAAT-3′). Agarose gel electrophoresis was performed to verify the size of amplicons. The SequalPrep Normalization Plate Kit (Invitrogen, Carlsbad, CA, USA) was used to clean up and normalize PCR products. Normalized amplicons were pooled in equal volume, and their quality and quantity were measured with Agilent Bioanalyzer 2100 (Agilent, Santa Clara, CA, USA) and quantitative RT-PCR, respectively. Illumina MiSeq 2 × 250 bp paired-end sequencing (MiSeq Reagent Kit v2, 500 cycles, 20% PhiX) was used to sequence pooled amplicons. Negative controls for DNA extraction and PCR amplification and mock community (ZymoBIOMICS™ Microbial Community Standard (Zymo, Irvine, CA, USA)) were included in each MiSeq run for quality control.

### Microbiome data analysis

Illumina MiSeq fastq reads were imported into the QIIME2 platform (version 2.4) and were processed by the Deblur program [15], which obtains single-nucleotide resolution based on error profiles within samples. Deblur denoised sequences are usually called amplicon sequence variants (ASVs), exact sequence variants (ESVs), or sub-operational taxonomic units (sub-OTUs). In this study, these sequences were assigned to bacterial features, which are synonymous to ASVs, ESVs, and sub-OTUs and sequences between different features differed at the single-nucleotide level. Deblur generates unique features that could be compared between different studies. To minimize the effects of sequencing depth on alpha and beta diversity measure, the number of reads from each sample was rarefied to 4000, which still yielded an average Good’s coverage of 97.90%. The taxonomy of these features was assigned to the Greengenes reference database (13-8 version) classifier with 99% similarity. A feature table was generated using Qiime2’s qiime vsearch cluster-features-closed-reference command. Determination of alpha and beta diversities and analysis of similarity (ANOSIM) were also conducted in qiime2. This data analysis pipeline yielded high-quality sequences as suggested by the eight bacterial taxa from the mock communities that were detected in each run with consistent relative abundance as expected.

Permutational multivariate analysis of variance (PERMANOVA) was performed to disclose the factors shaping the dynamics of the swine gut microbiome. PERMANOVA, a distribution-free algorithm, accommodates random effects, repeated measures, and unbalanced datasets [16]. For PERMANOVA analysis, we used the adonis function in the vegan package of R including different independent variables (e.g., age, gender, diet) with default settings (Bray-Curtis distance and 999 permutations). We used strata = pigID to account for the random effects of pigs for repeated measures. Stage-dependent features were identified by using the linear discriminant analysis (LDA) effect size (LEfSe) with default settings (e.g., LDA score > 2) [17]. Regression-based random forest models were developed to identify bacterial features that correlate with growth performance (body weight), using the default settings in the randomForest package in R project [18]. LEfSe was also used to identify bacterial features differentially represented between the control and the FMT groups in the validation trial.

The SparCC algorithm that is able to estimate the correlations from compositional network was used for network analysis. The network was demonstrated by using the igraph package in R with edges connecting nodes (bacterial taxa) with a correlation co-efficiency over 0.6 or less than − 0.6. Clusters were generated based on the betweenness centrality calculated with the Girvan-Newman algorithm [19].

### Growth performance data analysis

Data were analyzed using the general linear model of SAS (Cary, NC) as complete block design with treatment as a fixed effect. Each individual pig was used as the experimental unit. PDIFF option was used to test differences between least square means of the factor levels.

## Results

### The dynamics of the swine gut microbiome from birth to the market

We first characterized the dynamics of the swine gut microbiome in the test trial by analyzing a total of 273 rectal swabs collected from birth (d 0) to market (d 174). A total of 2,980,303 high-quality reads from 3358 features at the single-nucleotide resolution were generated with an average of 10,916 reads per sample. After rarefaction of sample reads to 4000, a total of 3254 features (1,080,000 total reads) from 270 samples were included for downstream analysis of the swine gut microbial community dynamics. The other three samples with sequence read number below 4000 were excluded from further analysis.

**Fig. 1:**
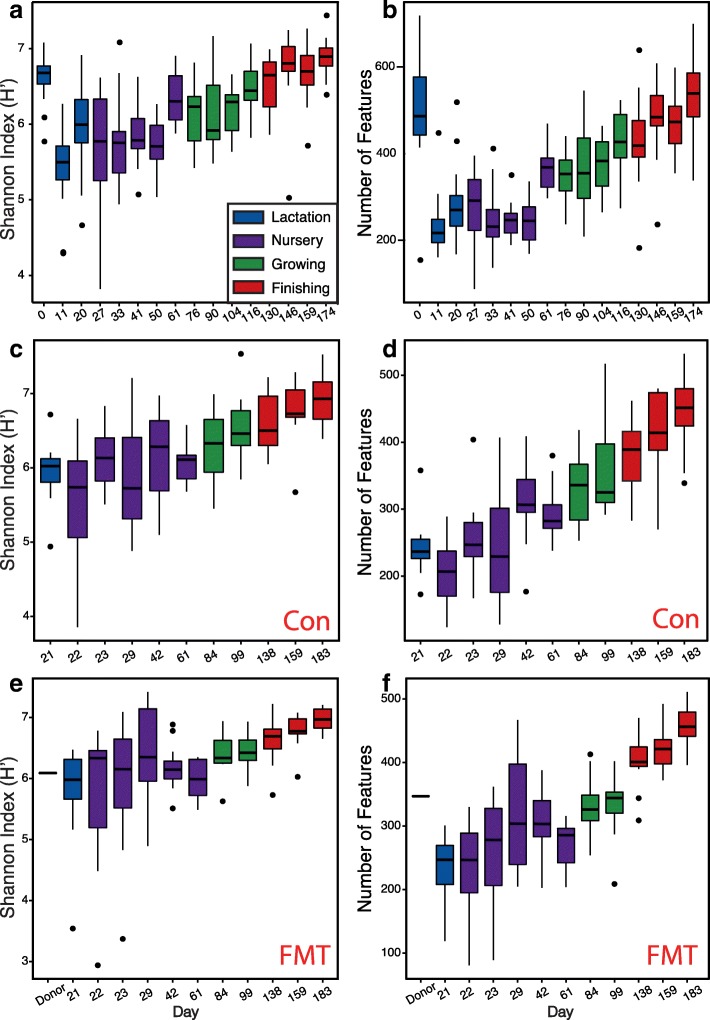
Longitudinal changes in the swine gut microbiome community diversity (**a**, **c**, **e**) and richness (**b**, **d**, **f**) from birth to market in the test trial (**a**, **b**), in the control group (**c**, **d**), and fecal microbiota transplantation (FMT) group (**e**, **f**) of the validation trial. Lactation, nursery, growing and finishing stages are depicted in blue, purple, green, and red, respectively

A high microbial diversity including the number of observed bacterial features and the Shannon index was observed in the meconium (d 0), comparable to the diversity of the adult pigs in this study (Fig. 1a, b), as well as to those of sows from other trials (Additional file 1: Figure S1a and b). The high microbial diversity dropped dramatically on day 11 and increased on day 20 before weaning. No significant changes in alpha diversity were observed during the first 4 weeks of the nursery stages, even though solid food was provided postweaning. The overall alpha diversity increased over time starting from the end of the nursery stage, as demonstrated by the Shannon index (H′, Fig. 1a) and the number of observed features (Fig. 1b) during the observation period (except d 0). No significant changes in community evenness were observed despite the slight increase in the finishing stage (Additional file 1: Figure S1c).

**Fig. 2:**
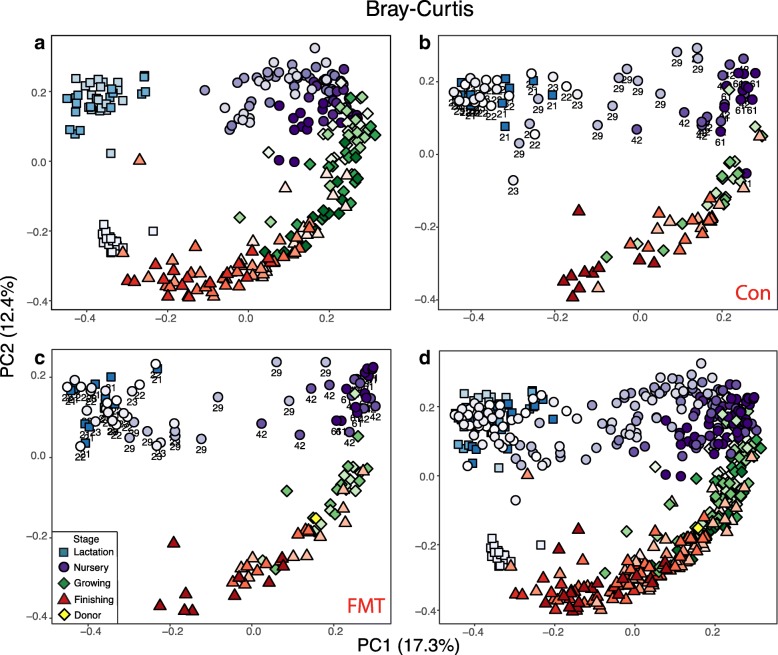
Longitudinal changes in the swine gut microbiome structure at different growth stages. Principal coordinate analysis (PCoA) plots based on the Bray-Curtis distances showed distinct clusters in the test trial (**a**), the control group (**b**), the fecal microbiota transplantation (FMT) group (**c**) of the validation trial, and all the groups combined (**d**). Lactation, nursery, growing, and finishing stages are differentiated by colors (blue, purple, green, and red, respectively) and shapes (square, circle, diamond, and triangle, respectively). The pig donor in the FMT group is indicated with a yellow diamond. Samples with same color densities were collected on the same day

Significant shifts in community membership and structure from lactation, nursery, growing, and finishing stages were observed on the principal coordinate analysis (PCoA) plots based on Bray-Curtis (Fig. 2a) and Jaccard (Additional file 1: Figure S2a) distances. Day 0 samples were distinct from those of the other two lactation time points (day 11 and day 20). The swine gut microbiomes were different between nursery, growing, and finishing stages when pigs were fed solid diets (Table 1 and Additional file 1: Table S2), but they were more similar to each other (ANOSIM, nursery vs growing: *R* = 0.425; growing vs finishing: *R* = 0.554, *P* = 0.001 for both) than to the lactation microbiomes when the pigs were fed sow milk (ANOSIM, *R* > 0.97, *P* = 0.001 for all solid feed stages vs lactation; Table 1 and Additional file 1: Figure S2a).

**Table 1:** Dissimilarities in the swine gut microbiome at different growth stages and meconium (d 0) revealed by analysis of similarity (ANOSIM) based on Bray-Curtis distances

Group 1	Group 2	Sample size	*R* value	*p* value	*q* value
Finishing	Growing	134	0.554	0.001	0.001
Finishing	Lactation	102	0.974	0.001	0.001
Finishing	Meconium	85	0.903	0.001	0.001
Finishing	Nursery	150	0.829	0.001	0.001
Growing	Lactation	102	0.991	0.001	0.001
Growing	Meconium	85	0.989	0.001	0.001
Growing	Nursery	150	0.425	0.001	0.001
Lactation	Meconium	53	0.905	0.001	0.001
Lactation	Nursery	118	0.981	0.001	0.001
Meconium	Nursery	101	0.995	0.001	0.001

### The “core” and stage-associated microbiomes

**Fig. 3:**
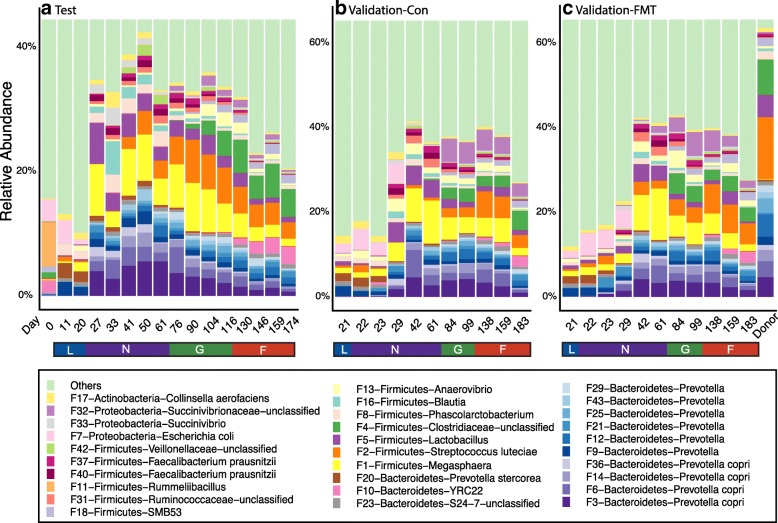
Top 30 features in the test trial (**a**), the control group (**b**), and the FMT group (**c**) of the validation trial. Each color represents the relative abundance of a bacterial taxon on the stacked bar chart.

We next examined the order and timing of bacterial taxa arrival during different stages of the pre-harvest section. At the phylum level, a total of 19 phyla including *Firmicutes*, *Bacteroidetes*, *Proteobacteria*, and *Actinobacteria* were observed, with *Firmicutes* being the most abundant phylum followed by *Bacteroidetes* across each stage (Additional file 1: Figure S3a). These two phyla accounted for 70% of the total sequences. At the sub-OTU level, the top 30 most abundant bacterial features are displayed on stacked bar charts. Among these top 30 taxa, 11 belong to genus *Prevotella*, the most diverse and dominant genus throughout most of the stages, especially after the introduction of solid feed (Fig. 3a).

**Fig. 4.**
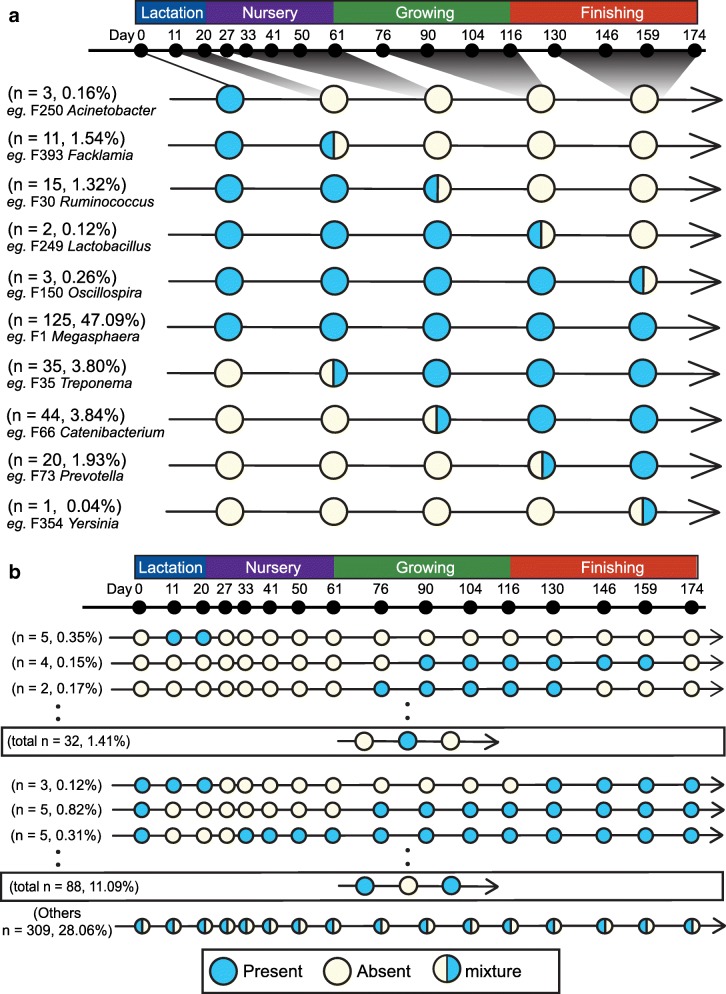
**a**, **b** Longitudinal occurrence patterns of the swine gut microbiomes. Top 700 features based on averaged relative abundance on each day were used to summarize the occurrence patterns. Blue circle indicates the presence of a bacterial taxon while a yellow circle shows the absence. Mixed color circles mean transition between “presence” and “absence” during each stage or time period

The appearance order and timing of the swine gut microbiome members are summarized in Fig. 4. Among the top 700 features, 125 features were present, based on their average relative abundance, throughout the entire pre-harvest lifetime and are defined as “core” microbiome or residents of the swine GI tract. Features that appeared only at certain stages are referred to as “stage-associated.” For instance, F250 (*Acinetobacter*) was observed only at birth (d 0). Feature 20 (*Prevotella stercorea*) was abundant during lactation but remarkably decreased in subsequent stages. Feature 7 (*Escherichia coli*) was present during the lactation stage and persisted till the end of nursery phase before phasing out. On the other hand, F3, which is associated with *Prevotella copri*, dramatically increased at the end of the first nursery phase after the introduction of solid food. Other features such as F4 (unclassified *Clostridiaceae*), 10 (*Bacteroidetes* YRC22), and 27 (*Clostridium butyricum*), which were rarely observed during lactation and nursery stages, increased rapidly during the growing and finishing stages (Fig. 3a and Additional file 1: Figure S4). Fluctuations in the relative abundance of bacterial features belonging to other dominant genera such as *Megasphaera*, *Lactobacillus*, and *Streptococcus* were also observed at various time points (Fig. 3a and Additional file 1: Figure S4). Finally, features that appeared sporadically at certain stages but disappeared later are called “passengers” (Fig. 4b).

**Fig. 5:**
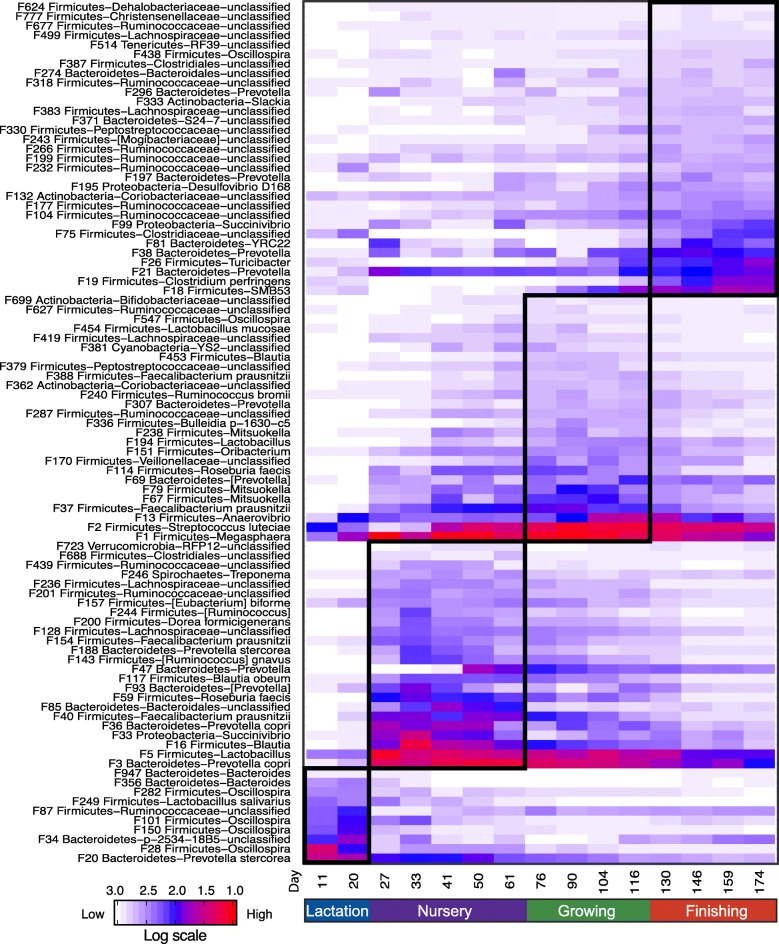
Heat map showing 91 stage-associated bacterial taxa identified by LEfSe (LDA > 2) in the test trial. The top 1000 features (d 0 samples were excluded) were used for LEfSe analysis. Heat map shows the average relative abundances on a log scale

Stage-associated bacterial features were identified by using LEfSe [17], an algorithm that focuses not only on statistical significance but also on biological consistency. The abundance of these features is visualized on a heat map (Fig. 5). Of note, day 0 samples were not included in the LEfSe analysis since meconium microbiomes were remarkably different from the typical lactation microbiomes on day 11 and day 20 and might bias the stage-specific data. LEfSe analysis confirmed most of the observations mentioned above. For example, F20 was classified as a lactation-associated bacterium, whereas F3 was nursery-specific, although both of these taxa belong to the *Prevotella* genus. Of note, although the core microbiome members persisted throughout the entire pre-harvest section, their presence also followed a stage-specific pattern. For example, *Megasphaera* (F1) and *Streptococcus luteciae* (F2) were detected starting d0 until the end of the study, but their abundance was relatively low during the lactation stage. Their abundance showed a unimodal pattern: increased starting nursery phase 1, peaked during the end of the nursery phase 3 and growing stage, and started to decrease thereafter (Fig. 5 and Additional file 1: Figure S4).

**Fig. 6:**
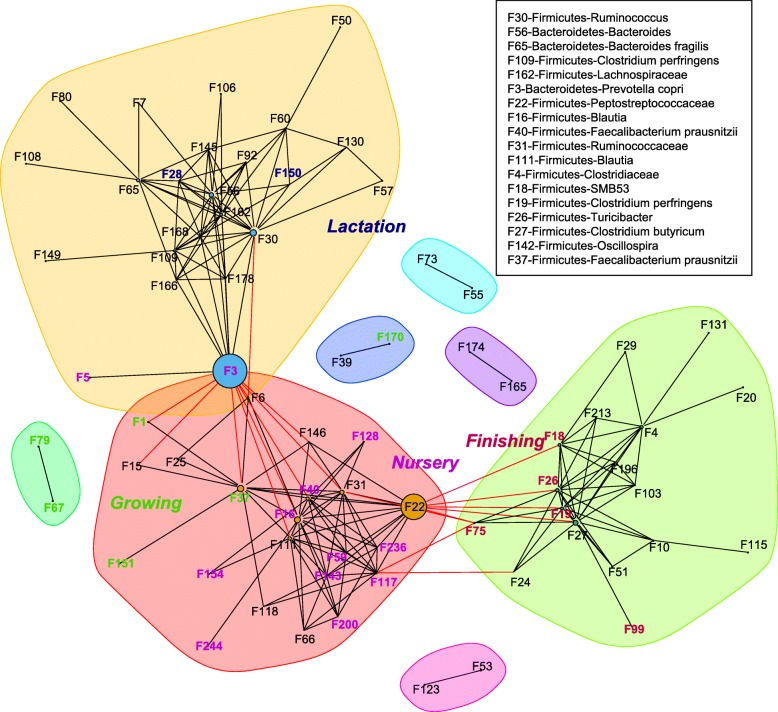
Network analysis of the interactions between bacterial taxa at different growth stages. SparCC was used to calculate the relationships between bacterial taxa. R package igraph was used to draw the network

Network analysis using the SparCC algorithm also showed stage-associated interactions between bacterial features (Fig. 6). Three large clusters within the network were observed with stage-associated features connected to one another. The yellow and green clusters connected nodes (bacterial features) associated with the lactation stage and finishing stages, respectively, whereas the pink cluster serves as a bridge connecting these two clusters by two hub nodes, F3 (*Prevotella copri*) and 22 (*Peptostreptococcaceae*). Bacterial features enriched in the nursery and growing stages were grouped in this cluster.

### Validation of the stage-associated swine gut microbiome

**Fig. 7:**
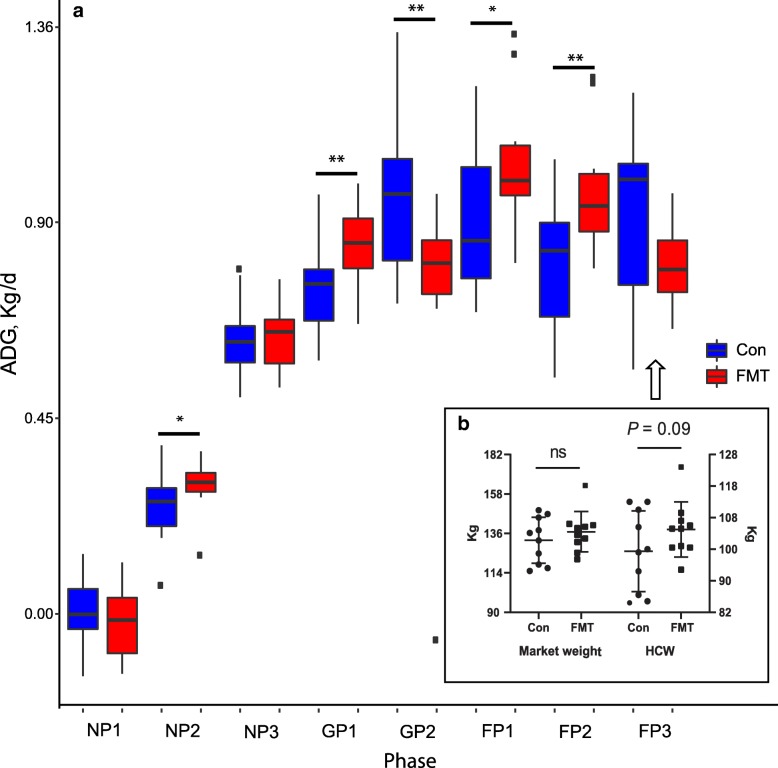
Effect of fecal microbiota transplantation (FMT) at weaning on **a** the average daily gain (ADG), and **b** final body weight and hot carcass weight (HCW) in subsequent stages of growth in pigs. All pigs were weighed at the beginning and the end of each phase to determine ADG and the market weight. At the end of this trial, all pigs were transferred to a plant where carcass characteristic data were collected. An asterisk (*) indicates a tendency for treatments significantly different; two asterisks (**) indicate traits significantly different

We next validated the stage-associated swine gut microbiome in a second animal trial (i.e., the validation trial). At weaning, we inoculated 12 pigs with mature gut microbiota isolated from a growing stage pig (growing phase 2). When compared to their littermates in the control group, FMT recipients had greater average daily gain (ADG) during nursery phase 2 (0.30 vs 0.25 kg, *P* = 0.087), growing phase 1 (0.86 vs 0.76 kg, *P* = 0.042), finishing phase 1 (1.05 vs 0.9 kg, *P* = 0.068), and finishing phase 2 (0.98 vs 0.81 kg, *P* = 0.018), but not at growing phase 2 (0.75 vs 0.96 kg, *P* = 0.042). Although not statistically significant, FMT pigs were 4.9 kg heavier at the end of the second trial and their hot carcass weight (HCW) was 7.7 kg heavier than their littermates (*P* = 0.09, Fig. 7).

Similar patterns in the development of the gut microbiome were also observed in the validation trial in both the control and the FMT groups. Alpha diversity sustained an increasing trend starting at weaning (d 21) until the end of the finishing stage in both groups (Fig. 1c–f). Introduction of solid food did not change the swine gut microbiome immediately. Microbiota on the first 2 days of the nursery stage (d 22 and 23) were still clustered with those collected from weaning (ANOSIM, *R* < 0.1, *P* > 0.05; Table 2). Significant changes in community structure were observed at the end of nursery phase 1 on d 29 after 8 days of solid feed consumption (Table 2, Fig. 2b, c), consistent with the animal trial 1 (Fig. 2a). In general, distinct clusters of the swine gut microbiome were observed in both individual animal trials and when combined together (Fig. 2d and Additional file 1: Figure S2d). Although FMT increased animal growth performance, it did not drastically change the swine gut microbiome (Additional file 1: Figure S5). Only minor changes in swine gut microbiome were observed on d 42 (ANOSIM, *R* = 0.24, *P* < 0.05) and 61 (ANOSIM, *R* = 0.16, *P* < 0.05).

**Table 2:** ANOSIM analysis of changes in the swine gut microbiome structures within the first nursery phase after solid feed supplementation

Group 1	Group 2	Sample size	Permutations	*R* value	*p* value
d21BT	d22BT	23	999	− 0.01	0.57
d21BT	d23BT	24	999	− 0.05	0.83
d22BT	d23BT	23	999	− 0.03	0.66
d21BT	d29BT	24	999	0.78*	< 0.01*

*indicates significant differences between two groups

As to the core microbiome, 147 and 125 features were identified from the control and the FMT group in the animal trial 2 among the top 700 features, respectively. Moreover, 69 of these core microbiome features were shared among three groups of pigs (trial 1, trial2—control, and trial2—FMT group) (Additional file 1: Figure S6). In addition, both the control (*N* = 31) and the FMT (*N* = 32) groups showed stage-associated swine gut microbiome features in the second trial (Additional file 1: Figure S7) as well. Among these features, two (F87 and F101), six (F16, F40, F59, F117, F128, and F143), four (F170, F114, F388, and F453), and three (F18, F195, and F333) features were shared between test trial and validation control groups at lactation, nursery, growing, and finishing stages, respectively.

### Diet shapes stage-specific swine gut microbiome

Permutational multivariate analysis of variance (PERMANOVA) was performed to elucidate the mechanism underlying the assembly of the stage-associated swine gut microbiome. Factors such as age, body weight, diet, gender, individual pigs, and sows were examined. Diet was used as a categorical variable including eight categories: lactation (sow milk), nursery (NP1, NP2, and NP3), growing (GP1 and GP2), and finishing diets (FP1 and FP2) in the test trial. We developed a series of models to determine the most important factors shaping the swine gut microbiome. We first performed PERMANOVA using univariate models. Diet, age, and body weight were all significant factors shaping the swine gut microbiome, with about 35% variation attributed to diet. Variability in individual pigs (PigID) also explained about 7% of variation in the swine gut microbiome, whereas gender and sows had little effect on the swine gut microbiome (Additional file 1: Table S3a). Given that age and body weight were highly correlated, we excluded BW in subsequent multivariate models. Diet was the most important factor in the multivariate model, with *F* value of 22.0 explaining about 35% of variation (Additional file 1: Table S3b).

In another multivariate model to determine which nutrients in the diet contributed most to the swine gut microbiome, we broke down the diets into different components including neutral detergent fiber (NDF), crude fiber, crude protein, and crude fat (Additional file 1: Table S3c). Since we did not measure the nutrients in sow milk, we excluded the lactation samples from subsequent models. Diet was still the most important variable in the new model, explaining about 34% of the variation (Table 3 (a)). NDF was the most important dietary nutrient in shaping the swine gut microbiome. Specifically, NDF from corn had the strongest effect with a pseudo *F* value of 66 explaining about 19% of the variation, followed by NDF from soybean (*F* = 20, *R*^2^ = 0.06) and DDGS (*F* = 10, *R*^2^ = 0.03) (Table 3 (b)).

**Table 3:** PERMANOVA analysis of the factors affecting the swine gut microbiome (multivariate models). Data were analyzed using R program Vegan package. Samples from nursery, growing, and finishing stages in the test study were used to perform PERMANOVA analysis with two sequential orders: diet, age, gender, sow origin, and PigID (a); NDF, crude fiber, crude protein, crude fat, age, sow origin, and pig ID (b)

(a)							
	Df	SumsOfSqs	MeanSqs	*F*	*R*^2^	*P*	Residuals
Diet	6	17.17	2.86	19.07	0.34	0.001	0.57
Age	1	0.48	0.48	3.20	0.01	0.004	
Gender	1	0.23	0.23	1.52	0.00	0.113	
Sow	2	0.64	0.32	2.13	0.01	0.006	
PigID	13	3.68	0.28	1.89	0.07	0.001	
(b)							
	Df	SumsOfSqs	MeanSqs	*F*	*R*^2^	Pr (> F)	
Corn NDF	1	9.90	9.90	65.97	0.19	0.001	
soybean NDF	1	3.02	3.02	20.10	0.06	0.001	
DDGS NDF	1	1.57	1.57	10.47	0.03	0.001	
Crude fiber	1	1.45	1.45	9.67	0.03	0.001	
Crude protein	1	0.30	0.30	2.00	0.01	0.037	
Crude fat	1	0.93	0.93	6.22	0.02	0.001	
Age	1	0.48	0.48	3.20	0.01	0.003	
Sow	2	0.67	0.33	2.22	0.01	0.003	
PigID (strata)	14	3.88	0.28	1.85	0.08	0.001	
Residuals	193	28.96	0.15	0.57			
Total	216	51.16	1.00				

Consistently, PCoA plot also shows the effect of these factors on the swine gut microbiome. Eight distinct clusters on the PCoA plot based on the Bray-Curtis distance assembled according to diet. No obvious clustering according to gender or sow was observed on the PCoA plots (Additional file 1: Figure S8). Although general succession patterns in the gut microbiome were observed in individual pigs, remarkable inter-pig differences in community structures were demonstrated at different stages (Additional file 1: Figure S9).

In addition, to assess whether priority effects, i.e., the order and timing of species arrival, play any roles in the assembly of the swine gut microbiota, we performed SourceTracker to measure the contribution of the early stage microbiome members to the later stage ones. A very small percentage of the swine gut microbiome was derived from early time points; only 3% of the lactation stage microbiome originated from the d 0 samples. Furthermore, the lactation stage microbiomes only contributed 8% to the nursery stage gut microbiome when the pigs were introduced solid feed. On the contrary, a remarkable percentage of the later stage microbiome originated from the nursery and growing stage microbiome when solid feed were consumed. The growing stage microbiome contributed 89% to the subsequent finishing stage, whereas 81% of its members originated from the nursery stage (Additional file 1: Figure S10).

### Growth performance-associated bacterial taxa within each stage

**Fig. 8:**
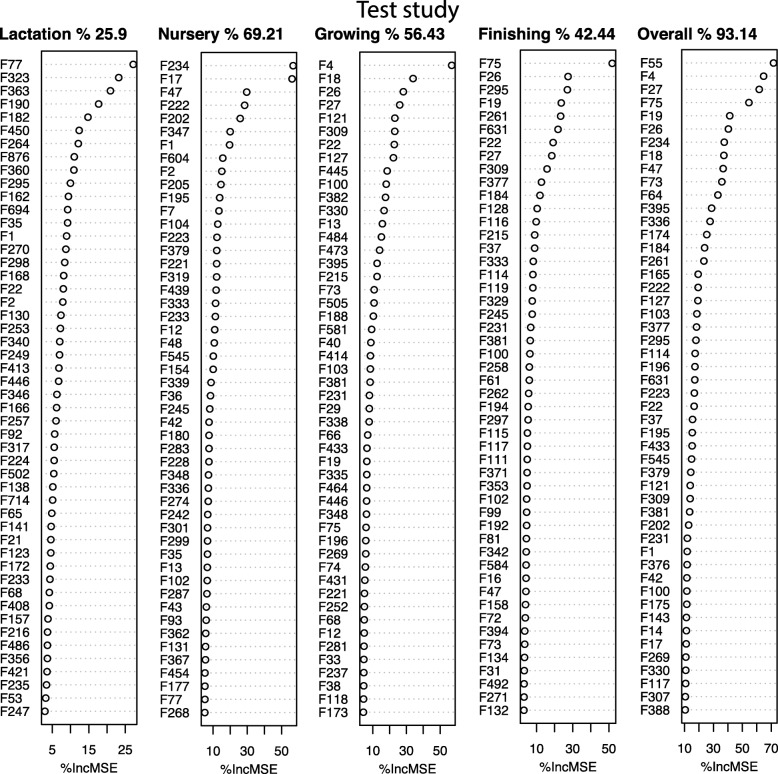
Growth performance-related features in the test trial. Top 50 growth-related bacteria at lactation, nursery, growing, finishing, and overall stages were selected from the top 500 features using regression-based random forest algorithm in R

We next sought to identify growth performance-associated bacterial taxa to be used as potential probiotics. To this end, we first performed regression-based random forest by using BW as the outcome and the top 500 bacterial features as predictors for each growth stage in the test trial. The top 50 bacterial features that predict growth performance at each stage are listed in Fig. 8. These features include members of both core and stage-specific microbiomes. For example, F1 and F2 were listed as growth performance-related features at the lactation and nursery stages, whereas F4 was a growing stage bacterium. Feature 27 (*Clostridium butyricum*), a butyric acid producer, together with F4 and F26 were positively correlated with BW at the growing and finishing stages. In addition, F18 and F19 were positively correlated with BW in older pigs (d 61-116 and d 116-174, respectively).

Interestingly, FMT from a growing stage donor did not change the swine gut microbiome at the nursery stage, i.e., the majority of the donor’s gut microbiome did not colonize in the recipients, but did enhance growth performance. A deeper analysis of the individual bacterial features by LEfSe identified several features enriched by FMT including F2 (only on d 42), F41, F454, F309, and F348 on d 42 and d 61 (Additional file 1: Figure S11). The relative abundance of these features increased at subsequent time points after FMT (Additional file 1: Figure S12). Notably, F2 and F454 are listed among top features correlated with BW (Additional file 1: Figure S13).

## Discussion

### Alpha diversity

An overall increasing trend in alpha community diversity and richness of the gut microbiome was observed during the pre-harvest lifespan of the pigs, consistent with previous studies [10, 12]. Increased richness and diversity from 10-day pre-weaning to 21-day post-weaning were shown in commercial pigs [12]. Community diversity (Shannon index) plateaued on d 146, whereas richness indices (number of observed features) kept increasing until the end of the experiment when the pigs were shipped for slaughter. The high alpha diversities on d 174 were very comparable to those of the sows, indicating a fully developed swine gut microbiome before market. In our study, we did not observe decreased gut microbiome diversity on the last sampling dates before market, which is inconsistent with previous reports. Han observed reduced alpha diversities starting on d 63 when antibiotics were supplied in diets [13]. In a recent study, De Rodas and colleagues [11] reported increased alpha diversity in different locations along the GI tract from birth to d 84. Interestingly, they also observed decreased diversity in market samples [11]. Of note, pigs in their study were fed antibiotic-free diets, like those in our trial, but were supplemented with pharmaceutical levels of zinc during the nursery stage. Therefore, the differences between these studies on alpha diversity could be due to high zinc levels. Human microbiome diversity increases from infancy to adulthood when the community matures and stays stable before decreasing as people age, likely as a result of changes in diet, dentition, medication, and physiology of gut ecosystems [20]. Although domestic pigs can live as long as 20 years, the pre-harvest pigs raised in this study and in most commercial farms are mainly grown for food production and are reared for 6–7 months only before slaughter. Therefore, it is difficult to determine when the swine gut microbiome plateaus and how the swine gut microbiome changes during aging. Nonetheless, gut microbiome diversity of endpoint pigs was comparable with those of the sows, suggesting that the swine gut microbiome matures after the finishing stage.

### Beta diversity: factors shaping the swine gut microbiome

This study provides a comprehensive view of the succession of the swine gut microbiome from birth to market by longitudinally collecting fecal samples from the same set of pigs across different growth stages. Such a study design allowed us to address several important ecological questions regarding the swine gut microbiome: (1) How does the swine gut microbiome change over time across different growth stages? (2) What are the underlying determinants of these changes?

Our study showed consistent patterns of succession of the swine gut microbiome along different growth stages among the three groups of pigs from two different animal trials. At birth, meconium samples showed greater community diversity than the other two lactation samples. Dramatic decreases in community diversity and significant changes in community structure were observed on d 11 and d 20 when the pigs were fed sow milk. Whether the in utero environment is sterile has been a controversial issue in the field of human microbiome area. The meconium samples were collected within 6 h after farrowing with possibilities for the pigs to suckle colostrum. Therefore, we cannot rule out the possibility of postnatal colonization of bacteria from the colostrum, sow teat skin, or the environment. However, given the fact that meconium samples were black and sticky, very different from other lactation fecal samples, and the meconium microbiomes were remarkably different from the day 11 and 20 fecal microbiomes, it is more likely that the meconium microbiomes were vertically transmitted from the sows rather than rapidly colonized by bacteria from other sources. Our study shows that, although the meconium bacterial biomass is low, with very low DNA concentration (less than 10 ng/μl), the meconium microbiomes are unlikely a result of contamination as they were distinct from those of the negative controls, and mock communities (Additional file 1: Figure S14). Thus, our data show that meconium samples harbor a diverse microorganisms (although with low bacterial load) that might serve as seeding bacteria to prime the development of the swine gut microbiome at subsequent time points or to educate postnatal innate and adaptive immune responses [21]. Colostrum and milk consumption is critical for the development of GI tract morphology, immune function, and the gut microbiome. Nutrients in milk such as oligosaccharides, amino acids, and fat activate digestive enzymes and chemical secretions, which alter the gut ecosystem for microbiome colonization [22]. The dramatic decrease of community diversity on d 11 indicates that the gut ecosystem during the first 10 days of life does not accommodate a highly diverse microbial colonization.

Another significant change in community structure occurred between d 20 (end of lactation) and d 27 (7 days postweaning), which might be attributed to weaning stress and/or the introduction of solid food. Weaning stress includes dietary and environmental transitions, and separation from the dam typically results in reduced feed intake and growth performance as well as a high incidence of diarrhea [23]. Stress has been reported to contribute to various degrees of microbial dysbiosis that affect immune and endocrine systems [24]. Diet serves as a major challenge during weaning transitions, causing both physical and metabolic reconstructions in the GI tract. Sow milk is highly palatable and digestible, whereas feed is rough, solid, less tasty, and not as easily digested [25]. Abrupt transitions to a solid feed diet induce short-term villus atrophy and crypt hyperplasia, which in turn impair digestive efficiency and gut integrity. A “leaky” gut could cause increased penetration of pathogens and nutrient loss.

Dramatic changes in both community structure and composition were observed 7 days postweaning. PCoA plots based on both Bray-Curtis and Jaccard showed distinct clusters completely separating the lactation and nursery microbiomes. Interestingly, such huge changes in microbiome did not happen in 1 day. In our validation trial, the microbiomes collected from the first 2 days (d 22 and 23) were not distinguishable from the end of lactation (d 21) samples. Given the fact that the d 27 samples in trial 1 and the day 29 samples in trial 2 were distinct from the d 20 end of lactation samples, we posit that it takes 7 to 9 days for the swine gut microbiome to adapt to a new diet and gut physiology.

The swine gut microbiome is driven by multiple factors such as host genetics, age, diet, environment, body weight, health, and antibiotics. Longitudinal studies are powerful given that animals serve as their own controls and many of the confounders are taken into consideration. However, it is also challenging in such studies to pinpoint which one is the major driver of the swine gut microbiome given that many of these factors are correlated. For instance, as pigs age, their body weights, rearing environments, and diet types also change accordingly. Therefore, we only selected age as a variable in the PERMANOVA models together with diet, sex, sow origin, and PigID. Diet was arguably the most important factor shaping the swine gut microbiome. Corn NDF in particular had the strongest effect in shaping the swine gut microbiome. NDF contains most of the structural components in plant cells such as lignin, hemicellulose, and cellulose that cannot be digested by the pigs and are consequently passed to the colon for fermentation by the swine gut microbiota. Our data is consistent with previous studies. Frese et al. reported that the GI tract catabolic pathways shifted from milk-derived glycan metabolism to plant glycan deconstruction and consumption after a solid feed diet was introduced to the pigs [26]. Our previous findings suggested that neonatal pigs provided with milk replacer along with solid diets during lactation had microbial community structures distinct from those of their sow-fed littermates, suggesting the significant effect of diet on the gut microbiomes of pigs of similar age [6]. Similarly, Bian et al. also pointed out that the impact of age and diet on gut microbiome succession surpasses that of sow genetics [27].

Age is another factor affecting the swine gut microbiome. Age is an indicator for physical maturation, which is accompanied by comprehensive functional transformations in metabolism, immunity, hormone secretion, muscle and bone development, and the nervous system [28, 29]. All these age-dependent biological alterations give rise to changes in microbiome structures [30–32].

### “Core” members, residents, passengers, and origins of the swine gut microbiome

This study also enabled us to address some other important biological questions regarding the swine gut microbiome, including (1) What is the core gut microbiome? (2) Which bacterial taxa are residents, persisting in the whole pre-harvest section from birth to market? (3) Which bacteria are passengers, present only at a certain point in time? (4) What were the origins of the swine gut microbiome (e.g., sows, diet, or environment)?

A total of 69 core microbiome members were shared between the three groups of pigs in the two animal trials, based on the definition that these bacterial taxa were present at least in one pig of each group at all the time points. Notably, a subset of these members (13 out of 69) was present in at least 50% of the pigs for at least 150 days from birth to market. These members include features 1, 5, 8, 9, 13, 17, 23, 46, 50, 62, 77, 112, and 132. Among these features, five (features 1, 5, 17, 62, and 132) were detected at all time points including d 0 (meconium), indicating vertical transmission of these bacterial taxa from the sows. These features were early colonizers of the swine gut and persisted throughout the entire pre-harvest lifespan, from sow milk-based lactation stage to the solid feed-based nursery, growing, and finishing stages.

Some new colonizers appeared and persisted after the introduction of solid feed. These features include features 3, 6, 12, 52, 63, and 153. Of note, F3 and F52 were detected in the d 0 samples as well, disappeared during lactation, then re-appeared after the solid feed supplementation in the nursery stage. Therefore, these taxa were likely vertically transmitted as well, but were suppressed during the lactation stage to an undetectable level, and proliferated when the nutrient and environment became more favorable. Many of these features belong to the genus of *Prevotella*, which was the largest genus in the swine gut microbiome at most of the time points during the solid feed stages. Members of *Prevotella* are associated with plant food-based diet and fiber digestion [33]. Interestingly, significant sub-OTU level differences in abundance and dynamics within this genus were observed. For example, members of *Prevotella copri* (features 3, 6, 14, and 36) proliferated during the nursery phase and gradually decreased at subsequent stages, whereas features of the unclassified *Prevotella* (e.g., F9) were one of the residents of the swine GI tract, present from lactation until the end of the finishing stage. The roles that *P. copri* plays in human health have been debatable. In a recent study, De Filippis et al. detected distinct strains of *P. copri* by metagenome studies and showed that diet might select distinctive *P. copri* populations [34]. Genes were enriched for drug metabolism in individuals on a Western diet, whereas genes in people consuming fiber-rich diets were enriched for complex carbohydrate degradation [34]. Introduction of solid fiber-rich feed during the nursery stage explains, at least partially, the increased abundance of *P. copri*. Similar to *P. copri*, members of *Megasphaera* (F1) and *Blautia* (F16) also increased postweaning, which is in agreement with previous reports [12]. Like *Prevotella*, members of *Megasphaera* and *Blautia* can also degrade carbohydrate efficiently [35–37]. Therefore, these microbes proliferated postweaning when pigs were provided with plant carbohydrate diets.

Later colonizers appeared during the late growing stage and persisted throughout the entire finishing stage. These late colonizers include features 4, 10, 18, and 19. Passengers refer to those bacterial taxa that showed up early or at the middle of the pre-harvest section but disappeared or faded out at later stages. Members associated with *E. coli* (F7) belong to the passenger category. In line with previous swine weaning [38–40] and human infant gut microbiome studies [41, 42], *E. coli* was abundant at birth (d 0) and during lactation stage but phased out after weaning, which could be due to the maturation of the immune system or suppression by other bacteria. Mucus presents a critical role for binding and preventing food-borne pathogens away from the host, and the physical structure of the mucosa is age-dependent [43, 44]. Pathogen-binding affinity of the mucus in immature animals is lower than in mature animals [45].

### Potential probiotics

The metabolic property of bacteria directly correlates with feed conversion rate and contributes to the host’s nutrient supply. Modulation of the gut microbiome to improve feed efficiency has become a novel strategy in the livestock industry. In our study, we identified top bacterial taxa that are most positively related to BW in adult pigs. Feature 26, associated with *Turicibacter*, was positively correlated with BW on d 90, 104, 116, 130, 159, and 174. Of note, *Turicibacter* is related to host immunity and is sensitive to host GI tract physiological conditions. *Turicibacter* populations were fewer in immunodeficient mice compared with their wildtype counterparts [46, 47]. Moreover, *Turicibacter* could reduce susceptibility to *Salmonella* infection in mice deficient in the expression of blood group glycosyltransferase β-1,4-N-acetylgalactosaminyltransferase 2 (*B4galnt2*), which is responsible for the synthesis of blood antigens. Hence, *Turicibacter* might play some positive roles in swine-microbiome immune interactions, consequently promoting an enhanced growth performance.

Feature 27 is a member of *Clostridium butyricum* (*C. butyricum*), with positive correlations with BW on d 130, 159, and 174; *C. butyricum* produces butyric acid, which serves as the most efficient energy source for livestock and GI epithelium maintenance. Dietary *C. butyricum* supplementation on weaning piglets has been reported to reduce the diarrhea score and enhance intestinal villus height [48]. Supplementation with *Butyricum* in grow-finishing pigs showed enhanced energy conversion rate [49]. Furthermore, *C. butyricum* is also involved in GI immunosuppressive modulation. Chen and colleagues showed that *C. butyricum* supplementation during weaning suppressed pro-inflammatory response indicated by increased mucosa IL-10 and reduced plasma tumor necrosis factor (TNF)-α [48]. Therefore, *C. butyricum* could enhance growth performance by providing more energy and/or improve the immune system. Features 4 and 18 are all associated with *Clostridiaceae*. These taxa proliferated in later stages (growing-finishing) and were positively correlated with BW. Of note, F4 was remarkably abundant (about 8% on d 174) at these stages. Features 4 and 18 were positively correlated with BW at almost all the last seven sample collection dates.

Features 2 (*Streptococcus*) and 454 (*Lactobacillus mucosae*) were identified as growth-related taxa during the nursery phase in the first animal trial. In the second validation trial, FMT did not significantly change the overall community structure but did improve animal growth performance. Interestingly, abundance of both of these two features was increased by FMT, suggesting the colonization of these features and their possible roles in promoting animal growth. *Lactobacillus mucosae* was first isolated from pigs with mucus-binding activity [50]. Members of this group have been reported to decrease epithelial permeability and improve barrier function. In another independent study, we detected improved growth performance in a group of pigs raised in an isolator with creep feed. In that study, F2 was also enriched in the high-performance group (Additional file 1: Figure S15) [6]. Although studies wherein these F2 strains are isolated and fed back to pigs would be necessary to prove their function in growth performance, our data in all these three trials corroborate F2 as a strong probiotic candidate.

## Conclusions

The swine gut microbiome has received growing attention due to the fact that pigs serve as an important protein source as well as an excellent biomedical model for human diseases. Despite the remarkable advances in our understanding of the swine gut microbiome from recent studies, many key ecological questions still remain unanswered. In this study, we characterized the longitudinal dynamics across all the different growth stages of the pigs in a test animal trial and validated these discoveries in a validation trial.

We observed consistent patterns of changes in swine gut microbiome structures along different growth stages in both animal trials. Diet, especially corn NDF, was the major driver of the swine gut microbiome. We identified 69 core microbiome members shared by the two animal trials. We also identified residents, passengers, early colonizers, and later colonizers of the swine gut. The order and time of species arrival, i.e., the priority effects, were more evident at later growth stages when solid feed were introduced. Although FMT did not significantly change the recipients’ gut microbiome, it did enrich a few bacterial taxa, which were correlated with increased growth performance.

Our study answered several of the key ecological questions in the swine gut microbiome and also provides a foundation for studies aimed at improving animal health and production.

## Additional file

Additional file 1: **Table S1-S3**, supplemental tables; **Figure S1-S15**, supplemental figures. (DOCX 4597 kb)

## Data Availability

The datasets generated during and/or analyzed during the current study are available in the Sequence Read Archive (SRA) repository, https://www.ncbi.nlm.nih.gov/bioproject/531671 (SRA accession #: PRJNA531671, available on May 30, 2019).
