# Supplementary material for: Longitudinal investigation of the swine gut microbiome from birth to market reveals stage and growth performance associated bacteria
Source: Microbiome. 2019 Jul 30;7:109. doi: 10.1186/s40168-019-0721-7 (PMC6664762; doi:10.1186/s40168-019-0721-7)
Supplement: Supplementary file 1 — Table S1-S3, supplemental tables; Figure S1-S15, supplemental figures. (DOCX 4597 kb) [file 40168_2019_721_MOESM1_ESM.docx]

Table S1. Number of fecal swab samples collected and used in data analysis in Trial 1 and 2.

| Animal trial 1 | |  |  |  |
| --- | --- | --- | --- | --- |
| Age (day) | Feed phase | Growth Stage | Number of samples |  |
| 0 | NA | Lactation | 18 |  |
| 11 | NA | Lactation | 18 |  |
| 20 | NA | Lactation | 17 |  |
| 27 | phase 1 | Nursery | 17 |  |
| 33 | phase 1 | Nursery | 17 |  |
| 41 | phase 2 | Nursery | 17 |  |
| 50 | phase 2 | Nursery | 16 |  |
| 61 | phase 3 | Nursery | 16 |  |
| 76 | phase 1 | Growing | 17 |  |
| 90 | phase 1 | Growing | 17 |  |
| 104 | phase 2 | Growing | 16 |  |
| 116 | phase 2 | Growing | 17 |  |
| 130 | phase 1 | Finishing | 16 |  |
| 146 | phase 1 | Finishing | 17 |  |
| 159 | phase 2 | Finishing | 17 |  |
| 174 | phase 2 | Finishing | 17 |  |
|  |  |  |  |  |
| Animal trial 2 | |  |  |  |
| Age (day) | Feed Phase | Growth Stage | #samples (control) | #samples (FMT) |
| 21 | phase 1 | Nursery | 12 | 11 |
| 22 | phase 1 | Nursery | 11 | 11 |
| 23 | phase 1 | Nursery | 12 | 12 |
| 29 | phase 1 | Nursery | 12 | 11 |
| 42 | phase 2 | Nursery | 12 | 12 |
| 61 | phase 3 | Nursery | 12 | 12 |
| 84 | phase 1 | Growing | 10 | 12 |
| 99 | phase 2 | Growing | 11 | 11 |
| 138 | phase 1 | Finishing | 10 | 11 |
| 159 | phase 2 | Finishing | 11 | 10 |
| 183 | phase 3 | Finishing | 10 | 10 |

Table S2. Analysis of similarity (ANOSIM) was used to determine swine gut microbiome dissimilarities between the four growth stages and meconium (d 0) in the test study based on the Jaccard distances.

| Group 1 | Group 2 | Sample size | Permutations | R | p-value | q-value |
| --- | --- | --- | --- | --- | --- | --- |
| Finishing | Growing | 134 | 999 | 0.600 | 0.001 | 0.001 |
| Finishing | Lactation | 102 | 999 | 0.995 | 0.001 | 0.001 |
| Finishing | meconium | 85 | 999 | 0.916 | 0.001 | 0.001 |
| Finishing | Nursery | 150 | 999 | 0.810 | 0.001 | 0.001 |
| Growing | Lactation | 102 | 999 | 0.999 | 0.001 | 0.001 |
| Growing | meconium | 85 | 999 | 0.986 | 0.001 | 0.001 |
| Growing | Nursery | 150 | 999 | 0.492 | 0.001 | 0.001 |
| Lactation | meconium | 53 | 999 | 0.907 | 0.001 | 0.001 |
| Lactation | Nursery | 118 | 999 | 0.981 | 0.001 | 0.001 |
| meconium | Nursery | 101 | 999 | 0.987 | 0.001 | 0.001 |

Table S3. PERMANOVA analysis of the factors affecting the swine gut microbiome. Data were analyzed using R program Vegan package. All 16 time points from d 0 to 174 in the test study were used to perform PERMANOVA analysis with univariate models (a) and multivariate model (b) with a sequential order of diet, age, gender, sow origin and PigID. Diet composition was listed in (c).

| a. |  | |  | | |  | | |  | |  |
| --- | --- | --- | --- | --- | --- | --- | --- | --- | --- | --- | --- |
|  | DF | SumsofSquares | | MeanSq | F.Model | | R^2^ | P | | Residuals | |
| Diet | 7 | 28.162 | | 4.023 | 20.518 | | 0.354 | 0.001 | | 0.646 | |
| Age | 1 | 8.969 | | 8.969 | 34.065 | | 0.113 | 0.001 | | 0.887 | |
| Bodyweight | 1 | 8.488 | | 8.488 | 32.331 | | 0.114 | 0.001 | | 0.886 | |
| Gender | 1 | 0.226 | | 0.226 | 0.764 | | 0.003 | 0.725 | | 0.997 | |
| PigID | 17 | 5.245 | | 0.309 | 1.047 | | 0.066 | 0.313 | | 0.934 | |
| Sow | 2 | 0.862 | | 0.431 | 1.4635 | | 0.011 | 0.049 | | 0.989 | |

| b. |  | |  | |  | |
| --- | --- | --- | --- | --- | --- | --- |
|  | Df | SumsOfSqs | MeanSqs | F.Model | R^2^ | Pr(>F) |
| Diet | 7 | 28.162 | 4.023 | 21.970 | 0.354 | 0.001 |
| Age | 1 | 1.353 | 1.353 | 7.390 | 0.017 | 0.001 |
| Gender | 1 | 0.206 | 0.206 | 1.127 | 0.003 | 0.245 |
| Sow | 1 | 0.380 | 0.380 | 2.074 | 0.005 | 0.021 |
| PigID | 16 | 4.936 | 0.309 | 1.685 | 0.062 | 0.001 |
| Residuals | 243 | 44.497 | 0.183 | 0.560 |  |  |

c.

|  | | | NP^1^ 1 | | NP 2 | NP 3 | GP^1^ 1 | GP 2 | FP^1^ 1 | FP 2 |
| --- | --- | --- | --- | --- | --- | --- | --- | --- | --- | --- |
| Ingredients, % of total diet | | | |  | |  |  |  |  |  |
| Corn | 38.8 | | | 44.4 | | 47.4 | 61.72 | 68.92 | 73.17 | 75.82 |
| Soybean meal, 48% | 25 | | | 31.6 | | 31.6 | 20.75 | 13.6 | 9.6 | 7 |
| Corn DDGS, >6 and <9% Oil | | 0 | | 15 | | 15 | 15 | 15 | 15 | 15 |
| Chemical composition | | | |  | |  |  |  |  |  |
| Total CP (%) | 22.9 | | | 25.4 | | 23.6 | 19.6 | 16.7 | 15.1 | 14.1 |
| Crude fat (%) | 5.4 | | | 6.6 | | 6.5 | 3.8 | 3.9 | 4 | 4.1 |
| Crude fiber (%) | 1.8 | | | 3.5 | | 3.5 | 3.4 | 3.2 | 3.2 | 3.1 |
| NDF^2^(%) | 5.6 | | | 11.2 | | 11.5 | 11.9 | 12 | 12 | 12.1 |
| NDF from Corn, % | 3.5 | | | 4.1 | | 4.3 | 5.6 | 6.3 | 6.7 | 6.9 |

1. NP: nursery phase; GP: growing phase; FP: finishing phase.
2. NDF: neutral detergent fiber

Figure S1. Longitudinal changes in swine gut microbiome community diversity (a) and richness (b) in sows and pre-harvest pigs that were followed from birth to market in the test trial. (c) shows the community evenness (Shannon Evenness) in the test trial. Lactation, nursery, growing, and finishing stages are based on dietary formation and physiological development, and are color-coded with blue, purple, green, and red, respectively. Sow rectal swabs, collected on farrowing day from another breeding group are coded in yellow.

Figure S2. Longitudinal changes in the swine gut microbiome structure during different growth stages. Principal coordinate analysis (PCoA) plots based on Jaccard distances showed distinct clusters in the test trial (a), the control group (b), the FMT group of the validation trial (c), and all the samples from the three groups combined (d). Lactation, nursery, growing, and finishing stages are labeled by colors (blue, purple, green and red, respectively) and shapes (square, circle, diamond, and triangle, respectively). Samples with same color densities were collected on the same day. The pig donor in the FMT group is indicated with a yellow diamond.


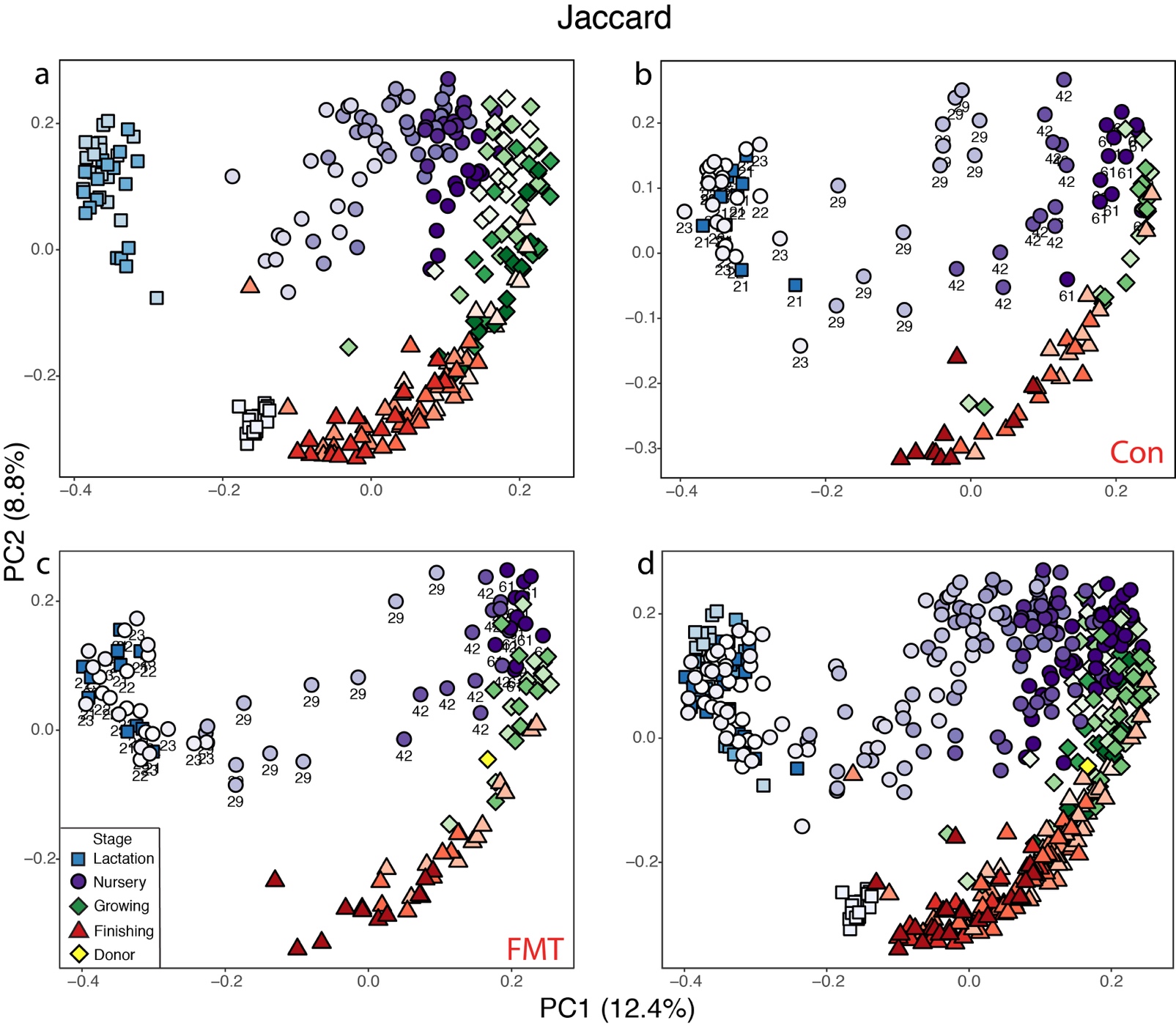


Figure S3. Stacked bar chart showing the longitudinal changes in the swine gut microbiome composition at the phylum level in the test trial (a), the control (Con) group (b) and the fecal microbiota transplantation (FMT) group of the validation trial (c).

Figure S4. Box plot showing the dynamics of the top 10 bacterial features in the swine gut microbiome of the test trial from birth to market.

Figure S5. PCoA plot showing the swine gut microbiome of the control and FMT groups in the validation trial. Lactation, nursery, growing, and finishing stages are distinguished by colors (blue, purple, green, and red, respectively). Samples with same color densities were collected on the same day. Con and FMT are distinguished by a triangle and a circle, respectively. Pig donor is indicated in yellow.

Figure S6. “Core” microbiome, identified as pre-harvest residents shared by test, validation-Con, and FMT groups.


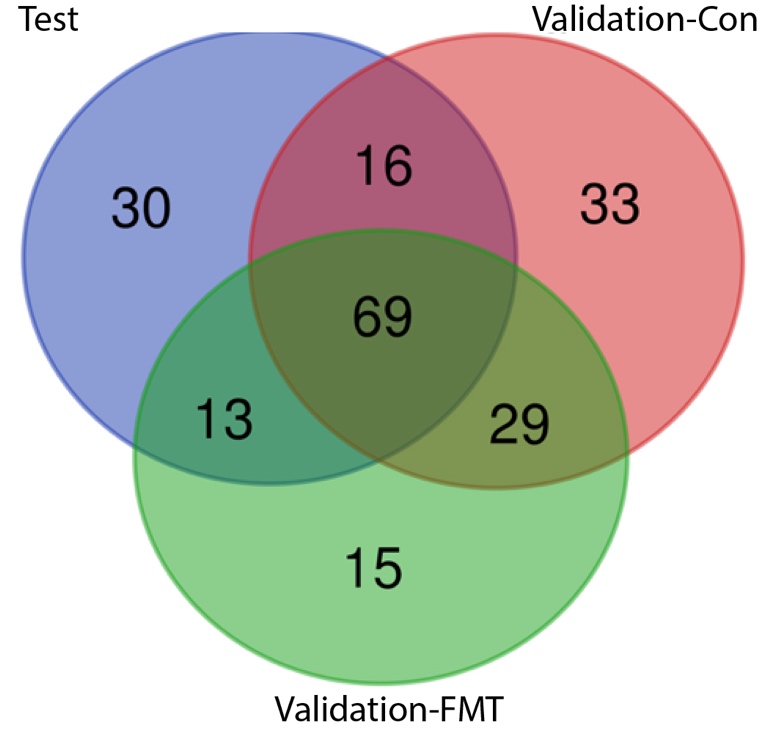


Figure S7. Stage-enriched features identified by LEfSe in control (a) and FMT (b) groups of the validation study based on the top 1000 features (excluding samples from d 22 and 23). Heatmap reflects averaged relative abundances on a log scale of stage-enriched features (LDA > 2) in the control (c) and FMT (d) groups with red indicating high abundance and blue indicating low abundance.

Figure S8. PCoA plots of longitudinal gut microbiome community in test trial, distinguished by diet, sow and gender. Lactation, nursery, growing and finishing stages are distinguished by shape (square, circle, diamond, and triangle, respectively).

Figure S9. PCoA plot (Bray-Curtis) of longitudinal gut microbiome for individual pigs with their ear notch number shown on each plot. Lactation, nursery, growing, and finishing stages were distinguished by shape (square, circle, diamond, and triangle respectively). Samples with same color densities were collected on the same day. Missing data or samples from d0 for certain pigs are not shown.

Figure S10. SourceTracker analysis of the contributions of the early-stage gut microbiome to the later-stage ones. The four box plots show the percentage of contribution from the d0 sample to d 11 (lactation), d20 (lactation) to d27 (nursery phase 1), d61 (end of nursery) to d76 (growing phase 1), and d116 (end of growing) to d134 (finishing phase 1).

Figure S11. LEfSe showing features differentially represented between the control and the fecal microbiota transplantation (FMT) group in the validation trial on d42 (a) and d61 (b). (c) shows the relative abundances of Feature 2 (second Y axis) and body weight (first Y axis) in the control (light blue) and the FMT (dark blue) groups at different time points.

Figure S12. Bacterial taxa enriched in the fecal microbiota transplantation (FMT) group at different time points in the validation trial. Blue and red bars represent the relative abundance of these bacterial features in the control and FMT group, respectively.

Figure S13. Growth performance-related features in the validation study. Top 50 stage-specific beneficial bacteria from lactation, nursery, growing, finishing, and overall stages were selected from the top 500 features in the test study using regression-based Random forest algorithm in R.


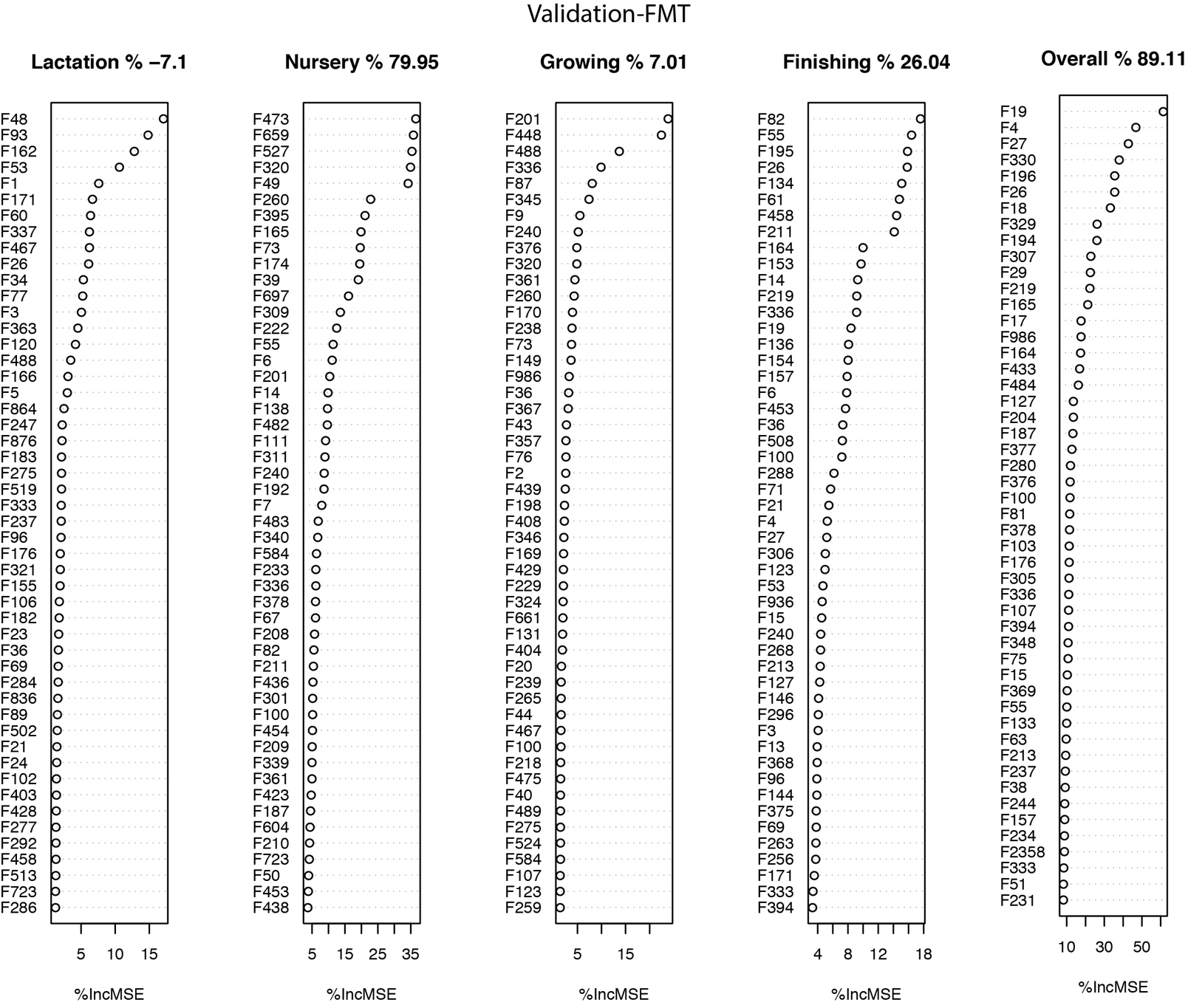


Figure S14. PCoA plot of negative controls, mock communities, and gut microbiome on day 0 piglets in the test study.

Figure S15. Relative abundances of F2 in control (Con) and isolated (ISO) groups on day d21, 64 and 78.
